# Crop Improvement: Now and Beyond

**DOI:** 10.3390/biology10050421

**Published:** 2021-05-10

**Authors:** Pierre Sourdille, Pierre Devaux

**Affiliations:** 1 INRAE, UMR 1095 INRAE—Université Clermont-Auvergne, Genetics, Diversity & Ecophysiology of Cereals, 5, Chemin de Beaulieu, 63000 Clermont-Ferrand, France; 2 Florimond-Desprez—Recherche & Innovation, 3 rue Florimond Desprez, P.O. Box 41, 59242 Cappelle-en-Pévèle, France

There is an urgent need to increase and improve the production of most agronomic species to meet the current food security challenge. Moreover, there are threats brought on by climate change as well as an increase in world population that is predicted to reach more than nine billion by 2050. Consequently, it has become crucial to accelerate the development of new and original high-yielding ideotypes. However, these ideotypes must simultaneously show better responses to biotic and abiotic stresses, including disease resistance, tolerance to drought, high temperatures, saline stresses, better nutritional value and greater flexibility in use, especially for industrial purposes such as biodegradable plastics or biofuels. In the meantime, it will be necessary to significantly reduce the environmental footprint of crop production; that means using less water, energy, fertilizers, pesticides, and fungicides, especially because some of these resources are vital, limited, or may present a danger to human health.

It is estimated that although about 2500 species began the process of domestication, only 10% were further improved for cultivation (for a review, see [1] and references therein). Domestication and diversification of crops are two processes that occurred roughly 6000–12,000 years ago (YA) in at least seven different regions of the world [1]. Domestication can be defined as the initial differentiation between wild ancestral species and the very first cultivated accessions that were consciously selected (e.g., involving traits such as seed or fruit size and non-shattering seeds). Diversification, rather, refers to further conscious improvements (e.g., more seeds or fruits). Because of the diversity of crops and the nature of the traits to be improved, many approaches need to be applied; some are specific to a crop (e.g., agronomical practices) while others—that are described below—are common to all of them.

First, the breeding of modern cultivars at the disposal of farmers has narrowed the genetic basis of crop species [2]. Diversity should therefore be re-enriched to create new innovative and high-performing cultivars under sustainable cropping conditions. Crop wild relatives (CWRs) are still present in natural habitats where they are able to develop adaptive traits to face environmental constraints; for example, this could be an efficient way to cope with drought tolerance [3,4]. CWRs have been exploited to improve crops for decades (for a review, see [5]). For example, *Oryza glaberrima* was used to improve cultivated rice (*O. sativa*; [6]), *Solanum pennellii* for tomato (*S. lycopersicum*; [7]), wild *Solanum* species for potato [8], wild *Gossypium* species for cotton (*G. hirsutum*; [9]) and *Aegilops ventricosa* for bread wheat (*Triticum aestivum*; [10]). However, there are several challenges in exploiting chromosome fragments from CWRs in crop genomes. The first is to carry out successful interspecific crosses since incompatibility between the CWRs and cultivated crops is common. Moreover, interspecific hybrids frequently undergo sterility due to disturbed meiosis, especially when species do not have the same ploidy level or because chromosomes are too divergent for an appropriate pairing. Proper chromosome pairing is a prerequisite for fertile gamete production. If not, reduced—or even an absence of—meiotic crossovers lead to spurious linkage drag between the genes controlling the traits of interest and those with negative agronomic effects. In addition, because CWRs cannot be easily sampled and evaluated under current agricultural practices, phenotypic data are only available for a few traits of interest, slowing down the wider use in breeding. All these barriers must be unlocked to utilize CWRs in breeding programs efficiently.

Second, because of the high number and complexity of domestication and diversification-related traits, numerous genome regions probably undergo selection pressure and are involved in the control of these traits. This simultaneously implies that thousands of genes are involved. For example, at least 500 regions (~2000 genes) within the maize genome were selected during the domestication process [11]. Identification of the best gene candidates involved in these traits is required to isolate alleles with differential effects, which could represent a positive response to various environments. Approaches searching for loci involved in quantitative traits (QTLs) have been applied to all crops and for many traits for more than 30 years, using molecular markers as landmarks. This has resulted in the positional cloning of numerous genes that can be further exploited by breeders for crop improvement. Moreover, the current development of new technologies relying on genome sequencing is paving the way for the cloning of many more genes of interest (for a review, see [12]). The cloning of candidate genes facilitates the discovery of new, original, and powerful alleles or their development through chemical (Ethyl-Methyl Sulfonate; EMS) or targeted (CRISPR) mutation. Additional efforts to identify relevant candidates in crops are thus needed to overcome a lack of knowledge that hampers the application of new strategies based on gene editing, or allelic diversity, for breeding purposes.

Third, for decades, breeding has relied on the collection of phenotypic data from parents and their progeny, resulting in breeding value estimates. During the 1990s, simulations concluded that a new approach of marker-based breeding (called Genomic Selection (GS)) combined with innovative multiplication technologies (doubled-haploidy, speed-breeding) to shorten the generation time might lead to a significant increase in genetic gain [13]. This concept simultaneously uses genetic values associated with DNA polymorphisms (usually single nucleotide polymorphisms (SNPs)) and phenotypic data; both collected from a training population to predict trait values without phenotyping breeding material. Moreover, this strategy can be used to work simultaneously with several quantitatively inherited traits from which even alleles with minor effects contribute instead of only a few major ones when marker-assisted selection (MAS) is applied. For example, since 2000, the development of low-cost high-density SNP arrays, with sometimes up to hundreds of thousands of markers, has transformed dairy cattle breeding, decreasing generation interval, and simultaneously doubling genetic progress for milk production (for a review, see [14]). Since then, similar approaches have been applied or evaluated for a wide range of plants (for a review, see [15]). However, much remains to be done, especially with regards to the development of high-density SNP arrays for some crops, for high-throughput phenotyping platforms for relevant agronomical traits, and accurate statistical models including linkage disequilibrium (LD), meiotic recombination, and genotype x environment (GxE) interactions, three key components of plant breeding.

Finally, the last decade has achieved a new paradigm with the amplification of revolutionary DNA/RNA sequencing methods, as well as the refining of single-cell, proteomic or metabolomic analyses. These new—omics approaches can now be integrated into breeding schemes (for a review, see [16]). However, the main question remains: what are the best traits to focus on? Responding to climate change appears to be the major challenge as increasing temperatures will concomitantly induce drought (and therefore salinity stress), sterility (because of the impact of high temperatures on meiosis), and the occurrence (or increased frequencies) of new pests and related diseases, which will undoubtedly result in yield and/or quality loss. These responses are collectively referred to as thermo-morphogenesis. Therefore, plants that can endure warmer temperatures are highly desirable, and progress has been reported lately [17]. For instance, in wheat, high temperature adversely affects plant development, productivity, and grain quality [18]. Nevertheless, since breeding considers all the traits simultaneously, all relevant results leading to better adaptation, resistance to any biotic or abiotic stress, and a higher yield would be helpful to breeders. The objective of this Special Issue is thus to show how many of these new tools have been developed and used for the improvement of major crops. It will also discuss how they will be used and improved in the coming years to meet the challenge of crop yield improvement to respond to human needs by 2050 in the context of sustainable agriculture.
